# Phytoplankton optical fingerprint libraries for development of phytoplankton ocean color satellite products

**DOI:** 10.1038/s41597-024-03001-z

**Published:** 2024-02-03

**Authors:** Michael W. Lomas, Aimee R. Neeley, Ryan Vandermeulen, Antonio Mannino, Crystal Thomas, Michael G. Novak, Scott A. Freeman

**Affiliations:** 1 Bigelow Laboratory for Ocean Sciences, East Boothbay, ME 04544 USA; 2 NASA Goddard Space Flight Center/Science Systems and Applications Inc., Greenbelt, Maryland 20771 USA; 3 NASA Goddard Space Flight Center, Greenbelt, Maryland 20771 USA; 4 Present Address: NOAA National Marine Fisheries Service, Silver Spring, Maryland 20910 USA; 5 Present Address: Institute of Carbon Cycles, Helmholtz-Zentrum Hereon, Geesthacht, Germany

**Keywords:** Marine biology, Microbial biooceanography

## Abstract

Phytoplankton respond to physical and hydrographic forcing on time and space scales up to and including those relevant to climate change. Quantifying changes in phytoplankton communities over these scales is essential for predicting ocean food resources, occurrences of harmful algal blooms, and carbon and other elemental cycles, among other predictions. However, one of the best tools for quantifying phytoplankton communities across relevant time and space scales, ocean color sensors, is constrained by its own spectral capabilities and availability of adequately vetted and relevant optical models. To address this later shortcoming, greater than fifty strains of phytoplankton, from a range of taxonomic lineages, geographic locations, and time in culture, alone and in mixtures, were grown to exponential and/or stationary phase for determination of hyperspectral UV-VIS absorption coefficients, multi-angle and multi-spectral backscatter coefficients, volume scattering functions, particle size distributions, pigment content, and fluorescence. The aim of this publication is to share these measurements to expedite their utilization in the development of new optical models for the next generation of ocean color satellites.

## Background & Summary

The Earth’s ocean ecosystems house a myriad of physical, chemical, and biological processes that create adaptive and resilient ecological communities of organisms in the sea. These ecosystems are an integral part of the planet’s biogeochemical cycles (e.g., carbon, nitrogen, phosphorus, silica, iron, etc.), which, in turn, are coupled to and influenced by the planet’s climate; the ocean’s biological carbon pump is one such cycle^1^. Of central importance to the biological carbon pump is phytoplankton functional type distribution and its response to changes in the physico-chemical environment, particularly those variables associated with climate change (e.g., ocean warming, increased stratification/nutrient depletion, and CO_2_ enrichment). For example, Lomas *et al*.^2^ showed that in the Sargasso Sea rapid warming during the decade of the 2010’s led to decreased net primary production, but the impacts on carbon export were mitigated by rapid changes in the phytoplankton community to favor small cyanobacteria and trophic processes that maintained carbon export rates. New generations of ocean ecosystem-biogeochemical models are integrating bio-optical data, such as what is presented in Neeley *et al*.^3^, to improve the representation of optically active components, including phytoplankton, which, in turn, will advance the representation of biogeochemical cycling^4,5^. The representation of phytoplankton communities in these models is key to linking ecosystem model outputs to ocean color data products in a changing ocean.

The need for state-of-the-art phytoplankton community composition (PCC) algorithms that derive some level of phytoplankton taxonomic biodiversity is critical to our understanding of how climate change is and will continue to impact oceanic food webs and carbon export. A variety of satellite algorithms to derive PCC or phytoplankton functional types have been developed over the last 10+ years. Most of these algorithms, as summarized in Mouw *et al*.^6^, derive relative abundances of phytoplankton size classes or particle size distributions from inherent optical properties (i.e., particle absorption or scattering properties), or total chlorophyll *a* (Chl*a*) derived from satellite algorithms. Size class information although useful for some applications, such as quantification of carbon export^7^, provides limited information about phytoplankton biodiversity, which can have important impacts on ocean biogeochemistry and the food web^8,9^. A limited number of algorithms use reflectance to either derive the dominant taxonomic group^10^ or the biodiversity of multiple taxonomic groups in the surface ocean^11^. An assumption of many of these algorithms is that Chl*a* is a reasonable proxy for phytoplankton biomass, conversion to other biomass metrics, such as carbon, is possible but requires additional derivative procedures (e.g.^12^). The merging of optical characteristics, Chl*a* and diagnostic pigment can generate a powerful tool to develop and validate algorithms for deriving phytoplankton size classes^13,14^.

The hyperspectral Ocean Color Instrument (OCI) on NASA’s Plankton, Aerosol, Cloud, ocean Ecosystem (PACE) mission will provide more opportunities to derive PCC at a higher level of taxonomic resolution. However, the PACE mission requires advanced algorithms to be developed for the hyperspectral ocean color data that will be generated. From the controlled laboratory conditions described in this manuscript, we have developed an optical fingerprint library for 50+ globally relevant phytoplankton taxa that may be used to develop these advanced algorithms for the determination of PCC. The advantage of this library is the inclusion of all optical properties, not just accessory pigments and Chl*a*, which may be used to distinguish challenging taxonomic groups that look similar from multispectral ocean color data. We provide some examples of how the data could be used, but this does not even scratch the surface of its potential applicability. An in-depth analysis of the pigment ratios and recommendations for use are fully described in Neeley *et al*.^3^.

## Methods

### Culture strains, culture methods, and experimental treatments

The strains chosen for this study (Supplemental Table 1) were cultivated at the National Center for Marine Algae and Microbiota (NCMA) at the Bigelow Laboratory for Ocean Sciences and are from regions where they are known to occur currently (e.g., diatoms from the North Atlantic, cyanobacteria from the oligotrophic gyres). Details of culture methods can be found in Neeley *et al*.^3^ and are only described briefly here. Cultures were grown axenically on a 14:10 L:D cycle at ~80 µmol photons m^−2^ s^−1^ for the warmer growth temperatures (>14 °C) and ~50 µmol photons m^−2^ s^−1^ at the cooler growth temperatures (<10 °C). All strains were grown using L1 medium^15^, except *Prochlorococcus marinus* that was grown in Pro99 medium^16^. While every practical attempt was made in this study to simulate the natural environmental conditions, we acknowledge that these are data from culture experiments and optical properties may differ from the same species in the natural environment. Direct quantification of taxonomically-resolved bio-optical properties under field conditions remains an area of additional research. *In vivo* Chl*a* fluorescence was measured to track biomass and determine the timing for use for experimental measurements. For *in vivo* Chl*a* fluorescence measurements, subsamples taken at the same time daily were analyzed on a calibrated TD-700 fluorometer^17^. Daily instrument response was tracked with a commercial solid fluorescence standard. Each stock culture was acclimated for approximately 10 divisions (~3 divisions/dilution cycle) before being scaled to 20 L and used for experimental conditions outlined below. Immediately prior to use in each experiment, cultures were diluted to a cell count that approximates their abundance in natural samples using Class A volumetric glassware. Unless otherwise noted, seawater from either the local Damariscotta River (Bigelow Laboratory, Maine; salinity of 32-33) or Sargasso Sea water were filtered to <0.2 µm for use as dilution water to produce the natural abundance cultures.

A range of experimental conditions were used to assess changes in cellular carbon, pigments and optical properties. For most of the strains, measurements were made at two growth stages: mid-exponential (Exp) and stationary (Stat) growth phase, but for some strains, measurements were made during only one growth phase. As phytoplankton populations in the field are rarely unialgal, a range of mixed cultures (mc) were made to provide data that can be used in the future to assess resolution of various algorithms.. The potential for changes in pigment composition in response to *in vitro* culture adaption over time (Evo) was assessed by studying multiple strains of the same species (*Amphidinium carterae*, *Ditylum brightwellii*, and *Heterosigma akashiwo*) isolated on different historical dates, but from similar geographic areas. *Synechococcus* sp., *Thalassiosira oceanica*, and *Ostreococcus lucimarinus* were examined for their response in pigment composition, cellular carbon, and optical properties to ‘climate change scenarios (CC)’ of elevated temperature, irradiance and decreased pH. All the various experiments conducted, and for which data are available, are noted in Supplemental Table 1 for the appropriate strains.

### Discrete measurements

#### HPLC pigments

Samples for pigment analysis were filtered onto glass fiber filters (~125 mm Hg), placed in aluminum foil pouches, flash frozen in liquid nitrogen before transfer to a −80 °C freezer. Phytoplankton pigment concentrations (Table 1, listed in order by retention time) were determined using well established high performance liquid chromatography methods^18,19^ and as described in detail in Neeley *et al*.^3^.

**Table 1:** Chromatographic and optical properties of phytoplankton pigments measured in this study in order of retention time in units of minutes (min) and absorption in units of nanometers (nm). Parentheses indicate a shoulder. The table was adapted from Neeley *et al*.^13^.

Pigment	Abbreviation	Retention time (min)	Absorption maxima (nm)
Chlorophyll *c*_3_	Chl*c*3	3.7	456, 588, (626)
Chlorophyll *c*_2_	Chl*c*1*c*2	5.9	446, 584, 634
Chlorophyll *c*_1_		6.2	442, 580, 632
Chlorophyllide *a*	Chlide*a*	6.3	(380), 434, 620, 666
Pheophorbide *a*	Phide*a*	8.1	410, 508, 538, 610, 666
Peridinin	Peri	10.0	476
19′ Butanoyloxyfucoxanthin	But	13.6	448, 468
Fucoxanthin	Fuco	14.0	452
Loroxanthin-like	Loro	14.9	(422), 446, 474
Neoxanthin	Neo	15.1	412, 436, 464
Prasinoxanthin	Pras	15.3	458
Violaxanthin	Viola	15.6	416, 440, 468
19′ Hexanoyloxyfucoxanthin	Hex	15.9	446, 468
Diadinoxanthin	Diad	17.2	(424), 446, 474
Alloxanthin	Allo	18.7	(428), 450, 480
Diatoxanthin	Diato	19.5	(428), 450, 478
Zeaxanthin	Zea	20.3	(428), 450, 476
Lutein	Lut	20.6	(422), 444, 472
Gyroxanthin diester	Gyro	23.4	(424), 444, 470
Divinyl Chlorophyll *b*	DVChl*b*	25.6	478, 606, 656
Monovinyl Chlorophyll *b*	Chl*b*	25.7	468, 602, 650
Divinyl Chlorophyll *a*	DVChl*a*	28.1	(390), 440, 624, 666
Monovinyl Chlorophyll *a*	Chl*a*	28.3	(388), 432, 618, 666
Pheophytin *a*	Phytin*a*	30.2	408, 506, 536, 608, 666
β,ε-carotene	Caro	31.2	(422), 444, 472
β,β-carotene	Caro	31.3	(428), 452, 476

Cellular Carbon to total Chl*a* (POC:Chl*a*) ratios were computed using the mean POC and Chl*a* concentrations from each growth phase or treatment. The errors associated with the separate analytical measurements of HPLC pigments and POC were propagated to POC:Chl*a* using standard error propagation theory^20^. The standard deviation of each average ratio was approximated using the equation:

1 $${\sigma }_{R}\cong \frac{{\mu }_{X}}{{\mu }_{Y}}\sqrt{C{V}_{X}^{2}+C{V}_{Y}^{2}+3C{V}_{Y}^{2}C{V}_{X}^{2}+8C{V}_{Y}^{4}}$$

where *σ*_*R*_ is the standard deviation of the ratio, *μ*_*X*_ and *μ*_*Y*_ are the average values of the pigment and carbon, respectively, *CV*_*X*_ is the coefficient of variation of the pigment and *CV*_*Y*_ is the coefficient of variation of the carbon measurement.

#### Particle and dissolved absorption

Replicate filter pads, two or three from each dilution, were collected for particle absorbance measurements by concentrating particles by vacuum filtration (~125 mmHg) onto either 25 mm Whatman GF/F filters or 25 mm GF75 filters (used for smaller cell sizes) using a glass filter cup and stem. Samples were placed in HistoPrep^TM^ tissue capsules, flash frozen in liquid nitrogen and transferred to −80 °C for storage until analysis. Measurements of filter pad particle optical density (OD_fp_) were performed using a Cary 4000 UV-Visible scanning spectrophotometer equipped with a 15 cm integrating sphere (Labsphere DRA-CA-900) following the protocol of Stramski *et al*.^21^ and further described in Neeley and Mannino^22^. Filters were moistened with 0.2 µm filtered, low dissolved organic matter seawater. The sample filter was placed on a plexiglass holder and jaw mount inside the integrating sphere chamber and measured at 0 and 90 degrees. Scans were performed between 290–850 nm with a 2 nm Slit Band Width (SBW), 0.2 nm data interval, reduced slit height and 120 nm per minute scan speed. Depigmentation of the filters was performed using the method of Kishino *et al*.^23^ and further described in Neeley and Mannino^22^, and analyzed as for the pigmented samples. The diameter of the filtered biomass was measured using calipers for computation of the pathlength. Blank filter scans were subtracted from the raw OD_fp_ spectra prior to *a*_p_ computation. Air scans were measured throughout the day to monitor instrument drift.

#### Dissolved organic carbon

Dissolved organic carbon (DOC) samples were collected from the filtrate of the particulate absorption samples in 40 mL amber glass vials (pre-cleaned from the manufacturer and combusted @450 °C for 6 h) and stored frozen (−20 °C) until analysis. For analysis, DOC samples were thawed and sonicated for 20 minutes in an ultrasonic bath. A Shimadzu TOC-L or TOC-V using the high temperature combustion catalytic oxidation method equipped with a total nitrogen unit was used to measure DOC concentrations^24,25^. The carbon standard potassium hydrogen phthalate (KHP) was used to generate calibration curves before each sample batch was analyzed on the instrument. Due to the broad range of DOC values, two five-point calibration curves were performed on approximately 2 mg/L and 4 mg/L KHP standards. Ultrapure water blanks (ultraviolet oxidized Milli-Q) were measured every three samples in the analysis queue to assess the instrument carbon blank, and the average water blank was subtracted from all sample values on a given analytical run. Several check standards of single concentration KHP were interspersed among samples in an analytical batch. Deep seawater consensus reference material (CRM; Rosenstiel School Hansell Organic Biogeochemistry Lab) was analyzed several times throughout each instrument sample batch to verify measurement accuracy. The carbon CRM materials measured with these samples were within reported values (CRM lot 10–17; 43.18 ± 1.07 uM C). The average concentration of carbon measured in the ultrapure water blanks throughout these analyses was 5.4 ± 1.39 uM C. Typical average percent coefficient between the measured and actual KHP was −1.4 ± 2.6%.

#### Particulate organic carbon and nitrogen

Samples for particulate organic carbon (POC) and nitrogen (PON) analysis, 50–100 mL depending upon the abundance of cells, were analyzed as described in Lomas *et al*.^26^. Briefly, samples were filtered onto 25 mm precombusted (450 °C, 5 h) Ahlstrom glass fiber filters and rinsed with 0.2 µm-filtered dilution seawater. Procedural blanks were created by rinsing a pre-combusted filter with an equal volume of filtered dilution seawater to account for any DOC adsorption^27^. All samples and blanks were frozen at −20 °C until analysis. Samples and blanks were dried at 60 °C, acid-fumed in a desiccator for 24 h, re-dried at 60 °C, and then analyzed on a Costech ECS 4010 CNS analyzer using acetanilide as a standard. Individual sample mass was corrected for the blank and converted to molar concentrations by dividing by the volume filtered.

#### Cell counts and biovolume

Samples for cell counts were collected at the time of sampling. Larger eukaryotic species were fixed with Alkaline Lugol’s solution (5% v/v) while cyanobacteria and *Ostreococcus* were fixed in freshly filtered (0.2 µm) paraformaldehyde (0.5% v/v). Lugol’s fixed samples were analyzed using a hemocytometer, counting at least 200 cells^28^. Cyanobacteria and *Ostreococcus* were counted by flow cytometry, using the volume analyzed method^29^. Biovolumes of larger cells were estimated from manual quantification of microscopy images, assuming a cylinder for diatoms where cell height in girdle view was assumed to be equal to the diameter, while the diameter was assumed to be equal to the height when in valve view. For all other cells, the volume was assumed to be that of an ovoid if the major and minor axes were different or a sphere when the axes were the same. Biovolumes of samples analyzed by flow cytometry were estimated by calibrated forward angle light scatter to beads and phytoplankton cultures of known diameters and assuming a spherical shape^30^. Direct quantification of detrital material was not conducted during sample analysis. Detrital particles and empty frustules in cultures of larger cells observed by microscopy were not common. A best qualitative assessment suggested that bias in organic biomass due to detrital particles was much less than 5%. For cultures analyzed by flow cytometry, particle events outside the cell population gate, after correcting for the sheath fluid blank, was ~5% and when converted to an estimate of biomass bias it was ~0.5% of cell biomass.

### Flow-through optics

#### Setup of flow-through optics

**Fig. 1:**
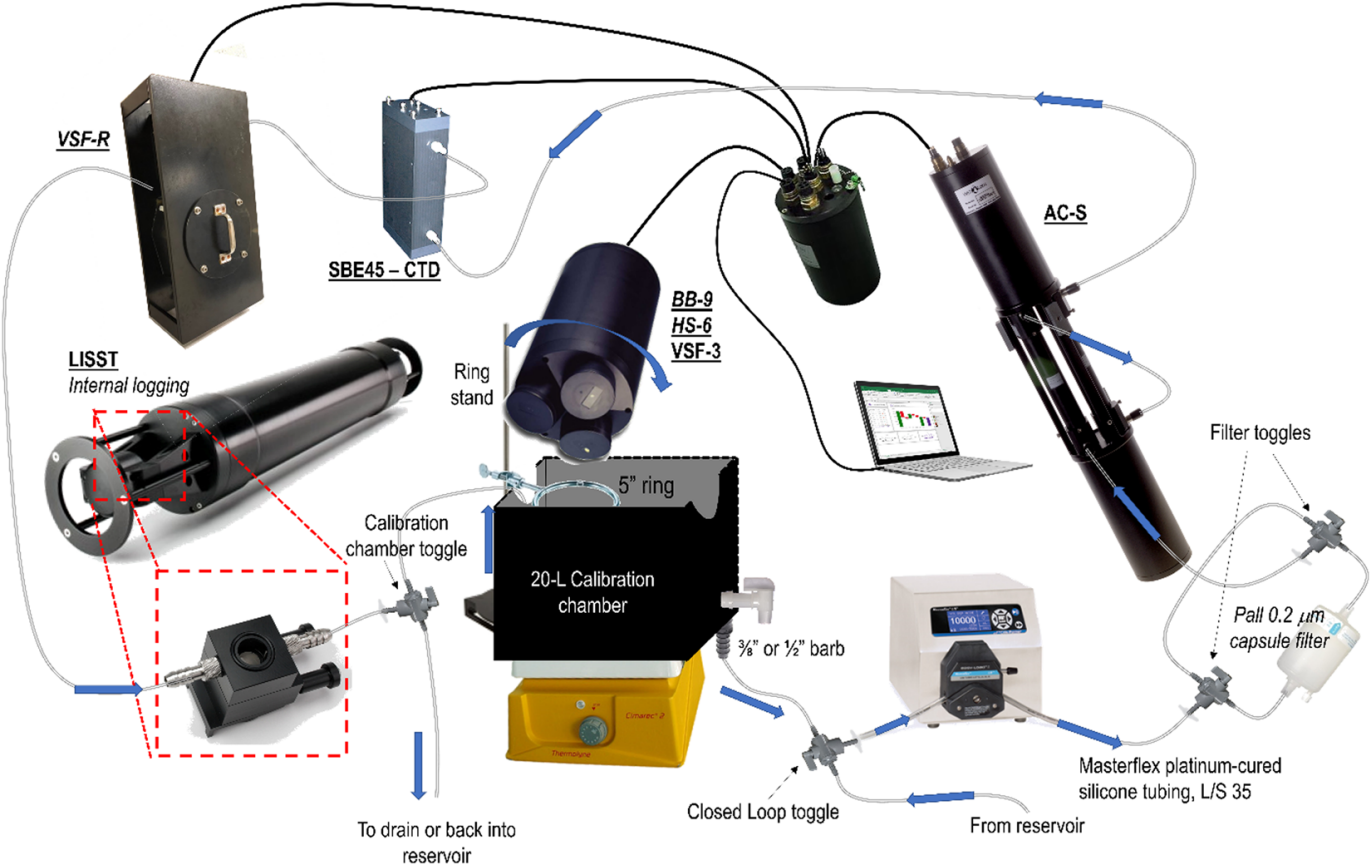
Schematic of in-line sampling apparatus for inherent optical properties of phytoplankton cultures.

A benchtop flow-through apparatus was constructed to measure the inherent optical properties (IOPs) of the diluted phytoplankton cultures (Table 2). While not all measurements were made on all phytoplankton strains, a set of core measurements from a WETLabs AC-S (hyperspectral absorption and attenuation meter on particulate samples), at least one backscattering instrument, and the LISST-100x were performed in duplicate for all strains. Upon homogenizing diluted cultures on a stir plate for a minimum of 10 minutes at 150 rpm, samples were pumped directly from the 25-L Nalgene carboys into a closed loop benchtop flowthrough system controlled by a Masterflex Easy-Load peristaltic pump in conjunction with Masterflex platinum-cured silicone tubing, L/S 35 (Fig. 1). Water flow was dynamically directed to the instruments through a series of 3/8” barbed three-way valves, terminating in a 20-L capacity black acrylic calibration chamber^31^. For some experiments, a larger volume chamber (black matte interior comparable to smaller chamber) was used to accommodate the larger HS-6 scattering instrument. When the terminal calibration chamber reached volume capacity with the contents of the Nalgene carboy, a return flow to the in-line system was initiated by switching a three-way toggle valve so that sample intake would be redirected from the carboy to the calibration chamber, closing the loop of the system. With the water looping through the in-line system, the optical instrumentation was gently tapped, and/or oscillatory pressure applied to the tubing to remove bubbles from the system. If necessary, flow rates were elevated on the peristaltic pump (up to 1.8 L min^−1^) to forcibly remove bubbles. Any volume lost to dislodging bubbles and removing air from the in-line system was subsequently replaced by toggling the three-way valve back to the carboy to demand more sample water.

**Table 2:** IOPs sampled by each specified instrument, over a defined range of wavelengths.

Measurement	Instrument	Wavelengths (nm)	Measurement Angles	Sampling Event
Particulate absorption, *a*_*p*_	WETLabs AC-S	400–730 (hyperspectral)	N/A	1–5
Dissolved absorption, *a*_*g*_	WETLabs AC-S	400–730 (hyperspectral)	N/A	1–5
Particulate beam attenuation, *c*_*p*_	WETLabs AC-S	400–730 (hyperspectral)	N/A	1–5
Dissolved beam attenuation, *c*_*g*_	WETLabs AC-S	400–730 (hyperspectral)	N/A	1–5
Particulate backscatter, *b*_*bp*_	HOBI Hydroscat-6 (HS-6)	375, 440, 488, 550, 620,700	141°	2–5
Particulate backscatter, *b*_*bp*_	WETLabs VSF-3	440,532,660	104°, 130°, 151°	1–5
Particulate backscatter, *b*_*bp*_	WETLabs VSF-R	650	104°, 130°, 151°	3–4
Particulate backscatter, *b*_*bp*_	WETLabs BB-9	409, 441, 488, 508, 526, 594, 652, 679, 717	124°	
Particle Size Distribution, *N(D)*	Sequoia LISST-100x	N/A	N/A	1–5
Temperature/Salinity	SBE45 MicroTSG	N/A	N/A	1–5

Also included are details on *b*_*bp*_*/VSF* measurement angles and sampling event in which specific instruments were used.

Once system equilibrium was achieved, the flow rate on the peristaltic pump was reduced to 0.5 L min^−1^, and optical measurements were made for a duration of at least 2 minutes to enable statistical exclusion of data anomalies/noise in post-processing. The larger, standalone scattering instrument (BB-9, HS-6, and VSF-3) measurements were performed sequentially in the 20-L calibration chamber, with the optical windows of the instruments submerged, and measuring at a distance of 18–20 cm above the bottom of the chamber^32^. In some experiments, a smaller, closed, in-line acrylic chamber was alternatively/additionally utilized for the backscattering measurements (using VSF-R sensor only; 650 nm), in order to reduce volume and time demands, and enable the characterization of a larger number of phytoplankton strains. After measuring the whole sample, a series of in-line filter toggles enabled the same water to be redirected to a 0.2 µm polyethersulfone cartridge filter prior to being re-introduced to the instrumentation, in order to assess the dissolved fraction of the IOPs. A separate dedicated 20-L black calibration chamber was used for measuring dissolved backscatter. The system was allowed to cycle through the filtration for at least 10 minutes at a rate of 1.0 L min^−1^, after the dedicated calibration chamber was filled prior to recording any measurements. Upon termination of sampling, all optical instrumentation was thoroughly cleaned on a daily basis with 70% ethanol and rinsed with Ultra-pure water (Milli-Q UV oxidized) and dried with Opti-Wipes^TM^. The tubing was flushed with at least 27-L of ultrapure water upon termination of sampling.

#### Pure water laboratory measurements and corrections

Pure water measurements were performed for all instruments daily. Ultrapure water (27-L) was collected daily and allowed to rest overnight to mitigate the impact of micro-bubbles on the measurement of inherent optical properties of the water. Following the same protocols as with sample water, the ultrapure water was introduced to the in-line IOP system through an in-line 0.2 µm cartridge filter by peristaltic pump, filling the 20-L acrylic calibration chamber before toggling the valve to loop the water through all instruments. For the AC-S, continuous measurements at discretized wavelengths were monitored over time, until all measurements > 450 nm were stable at ± 0.003 m^−1^ (inherent instrument noise). Measurements were compared with prior readings to ensure temporal stability over the course of sequential experiments. In addition to the ultrapure water blank measurements, dark counts on the backscatter instruments were collected by covering the optical windows with black vinyl electrical tape (3 M Scotch Super 33 + ) and submerging them in room temperature acclimated calibration chamber. A background scatter file for the LISST was collected in the z_scat_ chamber, following procedures outlined in the LISST-100x User Manual.

### Data Processing Procedures

#### Absorption and attenuation

All raw binary data were pre-processed with WET Labs Archive Processing program (WAP), which applies instrument specific calibration coefficients provided by the manufacturer, and temporally merges the IOP data with other data streams (e.g., temperature and salinity), outputting readable ascii files. Outlined below are the additional processing steps taken to apply daily ultrapure water calibrations, corrections for temperature, salinity, and the incomplete recovery of scattered light across the absorption tube pathlength. First, the median values from the ultrapure water calibration data were subtracted from the same-day data measurements of the non-fractionated and filtered ( < 0.2 µm) water samples. Temperature and absorption/attenuation salinity correction coefficients (*Ψ*_*T*_, *Ψ*_*Sa*_/*Ψ*_*Sc*_ respectively) provided from Sullivan *et al*.^33^ were then applied to the corresponding measured/reference temperature (*T*_*m*_/*T*_*r*_) and measured salinity (*S*_*m*_), and subtracted from the measured absorption (*a*_*m*_) and beam attenuation (*c*_*m*_):

2 $${a}_{t}(\lambda )={a}_{m}(\lambda )-[{({T}_{m}-{T}_{r})\Psi }_{T}]+[{S}_{m}{\Psi }_{Sa}]$$

3 $${c}_{t}(\lambda )={c}_{m}(\lambda )-[{({T}_{m}-{T}_{r})\Psi }_{T}]+[{S}_{m}{\Psi }_{Sc}]$$

Next, using beam attenuation values at a reference wavelength (λ _ref_ = 715 nm) with negligible influence from absorption, a scatter correction for the *a*-tube was applied from Zaneveld *et al*.^34^ to yield total, scatter-corrected absorption (*a*_*sc*_):

4 $${a}_{sc}\left(\lambda \right)=\left(\frac{{a}_{t}\left(\lambda \right)}{{c}_{t}\left({\lambda }_{ref}\right)-{a}_{t}\left({\lambda }_{ref}\right)}\right)\left({c}_{t}\left(\lambda \right)-{a}_{t}\left(\lambda \right)\right)$$

Finally, the particulate absorption (*a*_*p*_) and beam attenuation (*c*_*p*_) were derived by subtracting the median of the filtered absorption (*a*_*g*_) and beam attenuation (*c*_*g*_) data from the corresponding non-fractionated absorption (*a*_*sc*_) and beam attenuation (*c*_*t*_) data. Particulate total scatter (*b*_*p*_) was computed as the difference between *c*_*p*_ and *a*_*p*_:

5 $${a}_{p}\left(\lambda \right)={a}_{sc}\left(\lambda \right)-{a}_{g}\left(\lambda \right)$$

6 $${c}_{p}\left(\lambda \right)={c}_{t}\left(\lambda \right)-{c}_{g}\left(\lambda \right)$$

7 $${b}_{p}\left(\lambda \right)={c}_{p}\left(\lambda \right)-{a}_{p}\left(\lambda \right)$$

Median values of the measurement time series were reported to mitigate the impact of anomalous bubbles or inherent instrument noise. Values were interpolated to a consistent 2.5 nm sampling interval using a piecewise cubic Hermite interpolating polynomial function in MATLAB.

#### Backscatter and VSF

All raw binary data from the Wetlabs instruments (BB-9, VSF-3/VSF-R) were pre-processed with the WAP program to output readable ascii files, while HS-6 data were imported directly from the instrument software. As the first step in processing, the Wetlabs instruments angle and wavelength-specific scaling factors (*SF(θ,λ)*) and dark offsets (*DO(θ,λ)*) from the manufacturer were subtracted from raw counts (*V*):

8 $$\beta {\left(\theta ,\lambda \right)}_{t}=SF\left(\theta ,\lambda \right)\times \left[V\left(\theta ,\lambda \right)-DO\left(\theta ,\lambda \right)\right]$$

Next, a correction factor was applied to compensate for absorption across the pathlength between the light source and the detector on the backscatter instruments. The scatter-corrected absorption coefficient (*a*_*sc*_) from the AC-S was used to correct for absorption across the pathlength:

9 $$\beta \left(\theta ,\lambda \right)=\beta {\left(\theta ,\lambda \right)}_{t}\times {e}^{\left(\lambda \times {a}_{sc}\right)}$$

In these experiments, instead of computationally correcting for the volume scattering function of seawater^35^ as is common for *in situ* measurements where one cannot isolate the dissolved component, the values of β(θ,λ) obtained from the dissolved (<0.2 µm) measurements were subtracted from the unfiltered measurements to obtain the volume scattering function of the particle field only.

10 $$\beta {\left(\theta ,\lambda \right)}_{p}=\beta {\left(\theta ,\lambda \right)}_{unfiltered}-\beta {\left(\theta ,\lambda \right)}_{filtered}$$

For the BB-9 and HS-6 measurements, the particulate backscattering coefficient b(λ)_bp_ was estimated using a sampling angle-dependent (124° for BB-9, 141° for HS-6) volume scattering function conversion coefficient, or chi factor, χ^36^.

11 $$b{\left(\lambda \right)}_{bp}=2\pi \times \beta {\left(\theta ,\lambda \right)}_{p}\times {\rm{\chi }}$$

For VSF-3 and VSF-R measurements, each spectral measurement was collected at three different angles (*θ* = 104 °, 130 °, 151°), and the particulate backscattering coefficient at each wavelength was obtained by integrating *β(θ,λ)* in the backwards direction. First, the corrected *β*_*p*_ values were multiplied by 2πsinθ to convert to a polar steradian area. Then, a third order polynomial was fit to the three angular data points and a fourth datum of π radians = 0 (sin (π radians) = 0). Finally, an integration under the curve from π/2 to π radians yielded the *b(λ)*_*bp*_ coefficient. The median values of the *b(λ)*_*bp*_ time series were extracted in order to mitigate the impact of anomalous bubbles or inherent instrument noise.

#### Particle size and biovolume (LISST-100X)

A laser *in situ* scattering and transmissometer (LISST-100X Type-C, Sequoia Scientific, Inc.) was used to measure *in situ* particle size distribution and concentration. Detailed information of the LISST-100X operation is given in Agrawal and Pottsmith^37^. Clean filtered (<0.2 µm) water calibrations were performed daily. The scattering intensities measured by the LISST were post-processed using the manufacturer software, using a standard spherical inversion technique to obtain total particle volume concentrations (*PVC*) within 32 logarithmically spaced size classes from 2.5–500 μm (*d*). The particle number concentration, *N(D)*, was calculated as:

12 $$N\left(D\right)=\frac{PVC}{\left(\frac{4}{3}\right)\pi {\left(d/2\right)}^{3}}$$

To obtain an area size distribution, *A(D)*, the number of particles in each bin is multiplied by the average area of a particle in that bin, respectively.

13 $$A\left(D\right)=N\left(D\right)\times \left[\left(\pi /4\right){\left(d\times 1{e}^{-6}\right)}^{2}\right]$$

#### Particle absorption from the spectrophotometer

Absorption coefficients were computed using the method of Stramski *et al*.^21^. First, the optical density of suspended matter (*OD*_*s*_) was computed using Eq. 14.

14 $$O{D}_{s}=0.323{\left(O{D}_{fp}\right)}^{2.0867}$$

Next, total particle (*a*_*p*_) and de-pigmented particle absorption (*a*_*d*_) were computed using Eq. 15:

15 $${a}_{p,d}\left(\lambda \right)=ln\left(10\right)O{D}_{s}\left(\lambda \right)/\left(\frac{V}{A}\right)$$

where V is the filtration volume and A is the area of the particle load on the filter. The absorption coefficient of phytoplankton, *a*_*ph*_(λ), was computed as the difference between *a*_*p*_(λ) and *a*_*d*_(λ). Values are reported as the mean ± standard deviation of replicate measurements.

For a subset of the samples, pigment extraction was not 100% efficient, as evidenced by some pigment peaks remaining in the spectra, compromising the direct calculation of *a*_*d*_(λ). Therefore, smooth fits were performed to estimate *a*_*d*_(λ) on that subset of samples using the following three equations:

16 $${a}_{d}\left(\lambda \right)={a}_{d}\left({\lambda }_{0}\right)\exp \left[-{S}_{x}\left(\lambda -{\lambda }_{0}\right)\right]$$

17 $${a}_{x}\left({\lambda }_{0}\right)={a}_{x}\left({\lambda }_{0}\right){\left(\frac{\lambda }{400}\right)}^{{S}_{x}}$$

18 $${a}_{d}\left(\lambda \right)={a}_{d}\left({\lambda }_{0}\right)\exp \left[-{S}_{x}\left(\lambda -{\lambda }_{0}\right)\right]+k$$

where S defined the spectral shape of the curves, and *λ*_0_ was the reference wavelength at 400 nm. Equations 16 and 18 follow the exponential form in Roesler *et al*.^38^, while Eq. 17 follows the power law function from Twardowski *et al*.^39^. The initial values for the spectral shape in all equations were 0.012 and 0.014. Equation 18 includes a null correction, k, which was the average of the spectrum between 700–850 nm. For each sample, average values for S, determined via linear least-squares regression, were computed over the ranges 380–530 nm and 380–600 nm. The fit with the highest correlation coefficient and lowest root mean square error was retained and used.

## Data Records

All optical data for these 50+ phytoplankton strains are publicly available in Microsoft Excel^®^ XLXS format uploaded to Dryad^40^. The file is a multi-tab file with different data sources (e.g., particulate absorption from AC-S) on different tabs within the workbook. Each tab repeats the relevant metadata for each species to facilitate reference back to metadata. When possible, replicate analyses were conducted for each sample type. Here, in this paper, we report the mean values of the replicate samples. For all the sample data, “NA” indicate no data or not defined. All datasets are distributed under a CC0 1.0 Universal Public Domain Dedication license.

## Technical Validation

**Fig. 2:**
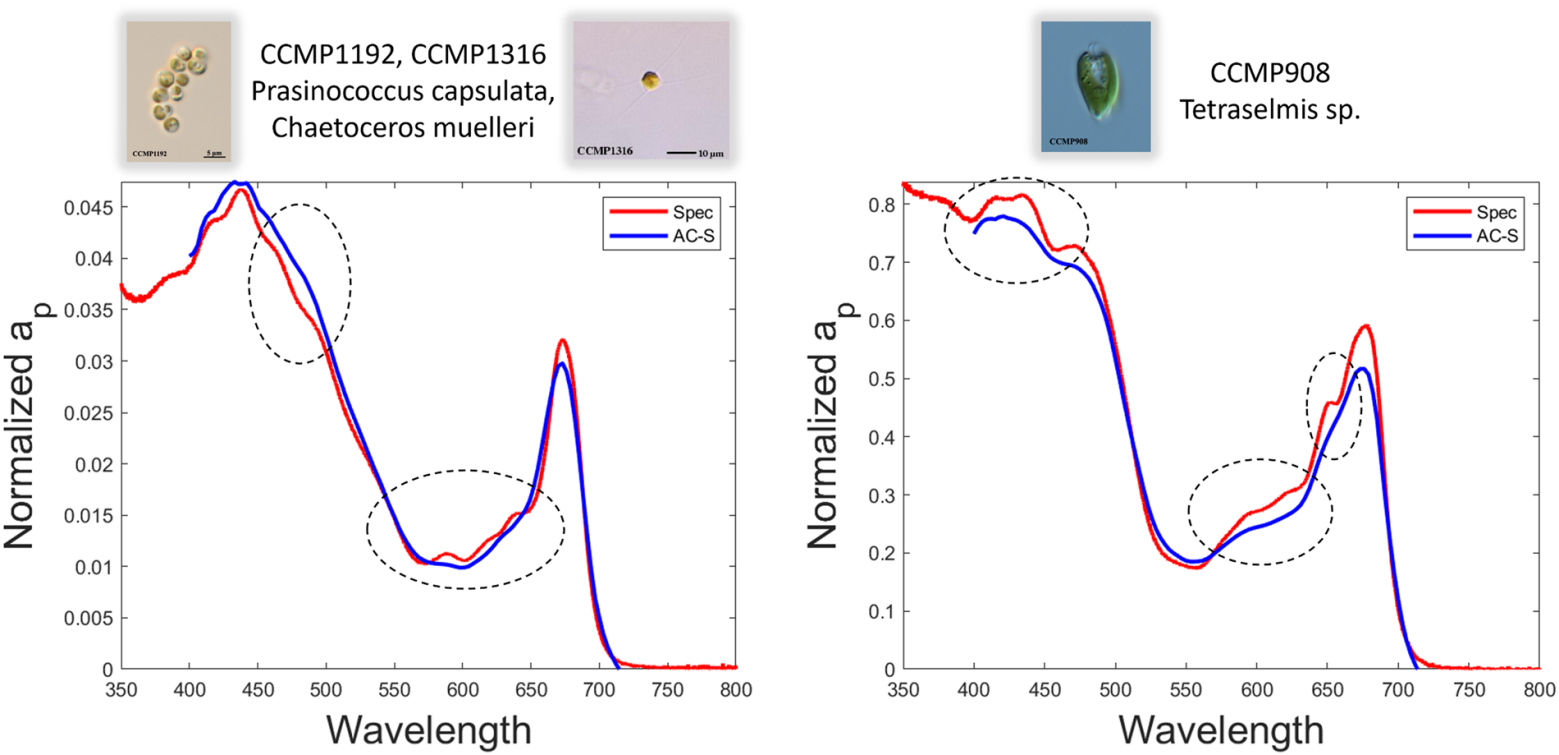
Comparison of particle absorption (a_p_) spectra derived from different instruments. Spectra were collected using a spectrophotometer (blue line) and an AC-S (red line) for a) mixed culture of *Prasinococcus capsulata* and *Chaetoceros muelleri* and b) unialgal culture of *Tetraselmis* sp.. Circled locations emphasize absorption peaks that are not resolved with the AC-S due to lower spectral resolution.

The data presented in this phytoplankton optical fingerprint library were all collected in a consistent manner across all strains. All cultures were grown under standardized conditions and diluted to cell concentrations that approximated *in situ* cell concentrations based upon published literature. All diluted cultures were homogenized and analyzed for a similar duration using well-vetted methods referenced above in the Methods section. Measurements of absorption were performed using a benchtop spectrophotometer via the filter pad method and an AC-S meter. Although both methods allow us to derive the absorption of particles, in this case pure phytoplankton monocultures, using the filter pad method, the absorption of de-pigmented materials can also be derived and used to compute the absorption by phytoplankton pigments alone. Additionally, the benchtop spectrophotometer provided higher resolution data (2 nm Slit Band Width, 0.2 nm data interval) compared to the AC-S data (14–18 nm Band Width, 3–5 nm interval). The advantage of the higher resolution attained with the filter pad method is that peak resolution is retained whereas some peaks in the AC-S data may be smoothed over owing to the lower spectral resolution (Fig. 2). Additionally, the spectral range of the spectrophotometric measurements reach further into the UV spectrum than the AC-S measurements (down to 290 nm in this study). The wider spectral range allows for the estimation of pigment and cellular absorption as well as the absorption by mycosporine-like amino acids (MAAs) that occur below 400 nm.

**Fig. 3:**
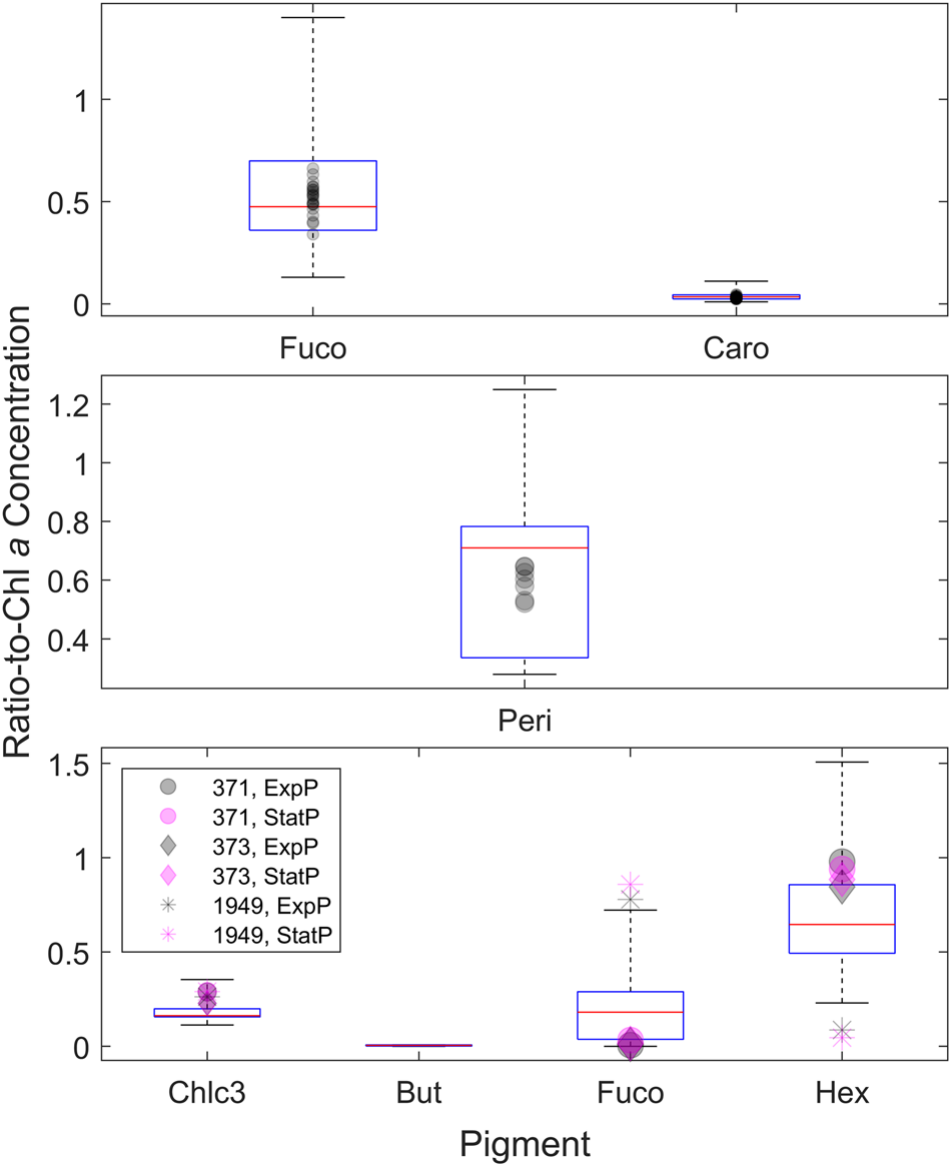
Box-whisker plots of pigment ratios normalized to Chl*a* for different taxonomic groupings. (**a**) dinoflagellates (**b**) diatoms and (**c**) *E. huxleyi*. Literature data are presented as the box-whisker plot and are overlain with the ratios determined in this study (scatter plots).

In Neeley *et al*.^3^, the pigment ratios derived from these experiments were compared to literature values for similar species. A literature review revealed a wide range of pigment ratios between species and within taxonomic groups. As observed in this study, and described in Neeley *et al*.^3^, interstrain differences within a species can occur, likely due to differences in the environmental conditions from which they were isolated. The pigment ratios determined from this study largely fell within the range of literature values (Fig. 3) for diatoms^32,41–48^ and peridinin-containing dinoflagellates^32,41,49,50^. In contrast, the strains of *E. huxleyi* diverged somewhat from literature values (Fig. 3c), potentially owing to the aforementioned differences in the oceanic regions from which they were isolated^41,44,51,52^.

Chlorophyll-specific absorption coefficients (a*_ph_) at five wavelengths determined from this study were compared to existing literature values from other culture studies^32,41,53–55^ Mean and standard deviation of a*_ph_(λ) for all species measurements within the major taxonomic groups were computed for this study and from the literature values. These values for four major taxonomic groups were reported at five wavelengths (443, 490, 500, 555 and 676 nm, Table 3). Generally, a*_ph_(λ) values determined during this study fell within range of those from the literature. Values for Prasinophytes from the literature were more variable but fell within range of those determined from this study,

**Table 3:** Mean and standard deviation (Sd) of a*_ph_ (λ;m^−1^) at five wavelengths from this study and from the literature values (Vaillancourt *et al*.^32^; Clementson and Wojtasiewicz^40^; Sathyendranath *et al*.^52^; Ciotti *et al*.^53^; Stuart *et al*.^54^).

Wavelength (nm)	Haptophytes		Diatoms		Dinoflagellates		Prasinophytes	
	PLOPS N = 19	Literature N = 6	PLOPS N = 21	Literature N = 18	PLOPS N = 10	Literature N = 8	PLOPS N = 9	Literature N = 9
	Mean ± Sd	Mean ± Sd	Mean ± Sd	Mean ± Sd	Mean ± Sd	Mean ± Sd	Mean ± Sd	Mean ± Sd
a*_ph_(443)	0.030 ± 0.012	0.033 ± 0.020	0.020 ± 0.009	0.028 ± 0.010	0.017 ± 0.003	0.022 ± 0.006	0.026 ± 0.015	0.053 ± 0.050
a*_ph_(490)	0.022 ± 0.010	0.016 ± 0.007	0.013 ± 0.005	0.020 ± 0.008	0.014 ± 0.002	0.018 ± 0.004	0.020 ± 0.009	0.038 ± 0.037
a*_ph_(550)	0.006 ± 0.002	0.005 ± 0.001	0.006 ± 0.001	0.009 ± 0.004	0.007 ± 0.002	0.009 ± 0.002	0.004 ± 0.002	0.008 ± 0.007
a*_ph_(555)	0.006 ± 0.002	0.004 ± 0.001	0.005 ± 0.001	0.008 ± 0.004	0.006 ± 0.002	0.008 ± 0.002	0.004 ± 0.002	0.008 ± 0.008
a*_ph_(676)	0.016 ± 0.005	0.015 ± 0.008	0.013 ± 0.005	0.017 ± 0.006	0.013 ± 0.002	0.013 ± 0.003	0.016 ± 0.005	0.030 ± 0.030

N is the number of data points used to compute the average values.

**Fig. 4:**
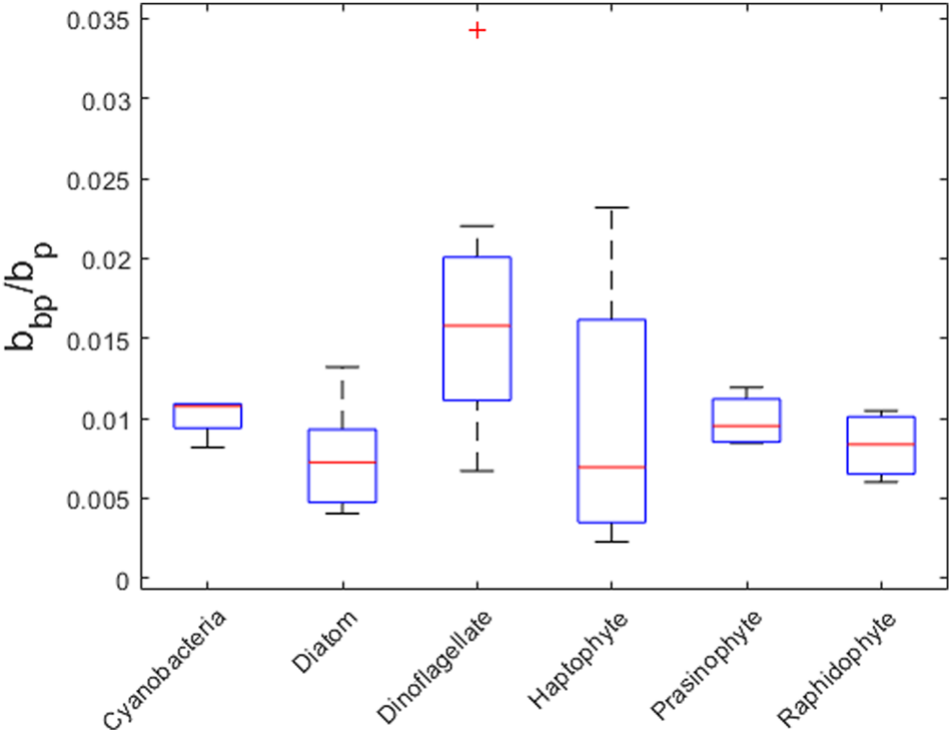
Backscatter ratio box-whisker plots for species examined in this study grouped by broad functional type.

Particulate backscatter coefficients (*b*_*bp*_) from this study were averaged by taxonomic group and compared with literature values (Fig. 4). These comparisons necessitate normalization since *b*_*bp*_ varies widely with cell density and biomass. Vaillancourt *et al*.^32^ measured scattering properties of 29 phytoplankton species, including *b*_*bp*_ at 4 wavelengths (440, 470, 510 and 620 nm), with a HS-6 instrument, similar to one of the instruments used in our study and reported *b*_*bp*_ normalized to chlorophyll-*a* concentration (*b*_bp_^*^(λ)), as well as *b*_*bp*_ normalized to POC and cell abundances. With the exception of the Haptophyte group, the *b*_bp_^*^(λ) values from the two studies matched quite well (Table 4). We examined Haptophyte strains with and without liths (Supplemental Table 1), whereas Vaillancourt *et al*.^32^ examined three strains (*I. galbana*/CCMP1323; *Chrysomulina polylepis*/CCMP1757; *Pavlova* sp./CCMP616), only one of which was characterized in this study. Backscattering measurements of *I. galbana* from our study were performed only in flow-through mode, but those *b*_bp_^*^(λ) values are comparable to measurements reported by Vaillancourt *et al*.^32^. *b*_bp_^*^(λ) values for Rhaphidophytes and cyanophytes in our study compared most closely to values in Vaillancourt *et al*.^32^. Both studies examined the same species of Raphidophytes, *Heterosigma akashiwo*, and the same strain (CCPM452) plus several other strains in our study. Vaillancourt *et al*.^32^ examined one Cyanophyte, *Synechococcus elongatus*, while this study interrogated two different *Synechococcus* species and *Prochlorococcus marinus*. Other studies investigating scattering properties of phytoplankton cultures have quantified *b*_*bp*_(λ), but normalized values were either not provided or not included in tabulated format to allow for intercomparisons^56,57^.

**Table 4:** Comparison of spectral particulate backscatter coefficient normalized to chlorophyll-*a* concentration (b_bp_*) from this study with literature values listed by taxonomic group.

Taxon	b_bp_*(440) (m^2^ mg Chl-*a*^−1^) ^1^	b_bp_*(440) (m^2^ mg Chl-*a*^−1^) HS6 ^2^	b_bp_*(440) (m^2^ mg Chl-*a*^−1^) VSF-3 ^2^	b_bp_*(441) (m^2^ mg Chl-*a*^−1^) BB9 ^2^	b_bp_*(510) (m^2^ mg Chl-*a*^−1^) ^1^	b_bp_*(508) (m^2^ mg Chl-*a*^−1^) BB9 ^2^	b_bp_*(620) (m^2^ mg Chl-*a*^−1^) ^1^	b_bp_*(620) (m^2^ mg Chl-*a*^−1^) HS6 ^2^
**Chlorarachniophyte (Sd)**	1.14E-03 (1.29E-03)	5.4E-04 (na)	1.3E-03 (6.6E-04)	na (na)	1.00E-03 (1.11E-03)	na (na)	8.66E-04 (1.01E-03)	7.0E-04 (na)
**Cryptophyte (Sd)**	1.56E-03 (2.05E-03)	9.3E-05 (6.8E-05)	1.8E-04 (5.9E-05)	2.2E-04 (8.7E-05)	1.15E-03 (1.42E-03)	2.0E-04 (2.8E-05)	9.90E-04 (1.11E-03)	1.1E-04 (1.6E-05)
**Cyanophyte (Sd)**	9.65E-04 (na)	7.1E-04 (4.0E-04)	2.1E-03 (3.7E-03)	1.2E-03 (5.7E-04)	7.16E-04 (na)	9.9E-04 (4.3E-04)	5.18E-04 (na)	5.4E-04 (3.4E-04)
**Diatom (Sd)**	7.74E-04 (4.35E-04)	1.5E-03 (3.0E-03)	2.0E-03 (4.2E-03)	1.9E-03 (3.8E-03)	5.30E-04 (2.79E-04)	1.6E-03 (3.3E-03)	4.06E-04 (2.06E-04)	1.0E-03 (2.3E-03)
**Dinoflagellate (Sd)**	1.65E-03 (1.8E-03)	5.8E-04 (5.4E-04)	7.7E-04 (5.7E-04)	8.2E-04 (5.8E-04)	1.25E-03 (1.5E-03)	7.9E-04 (5.2E-04)	1.27E-03 (1.64E-03)	4.5E-04 (3.6E-04)
**Haptophyte (Sd)**	6.54E-04 (1.48E-04)	7.8E-03 (1.4E-02)	6.7E-03 (1.4E-02)	1.7E-02 (2.1E-02)	4.82E-04 (4.08E-05)	1.1E-02 (1.4E-02)	4.15E-04 (3.79E-05)	5.3E-03 (9.1E-03)
**Pelagophyte (Sd)**	2.38E-03 (2.92E-03)	na (na)	9.1E-03 (3.4E-03)	na (na)	1.61E-03 (1.86E-03)	na (na)	1.38E-03 (1.63E-03)	na (na)
**Prasinophyte (Sd)**	1.57E-03 (1.00E-03)	8.9E-04 (7.1E-04)	7.0E-04 (8.6E-04)	2.6E-03 (3.5E-03)	1.43E-03 (9.07E-04)	5.9E-03 (1.0E-02)	1.11E-03 (7.95E-04)	5.7E-04 (2.8E-04)
**Raphidophyte (Sd)**	3.13E-04 (na)	1.9E-04 (2.6E-04)	1.6E-04 (2.3E-04)	1.8E-04 (2.3E-04)	2.39E-04 (na)	1.5E-04 (1.9E-04)	2.06E-04 (na)	1.1E-04 (1.4E-04)

^1^Vaillancourt *et al*.^32^ and personal communication; ^2^This study.

## Usage Notes

**Fig. 5:**
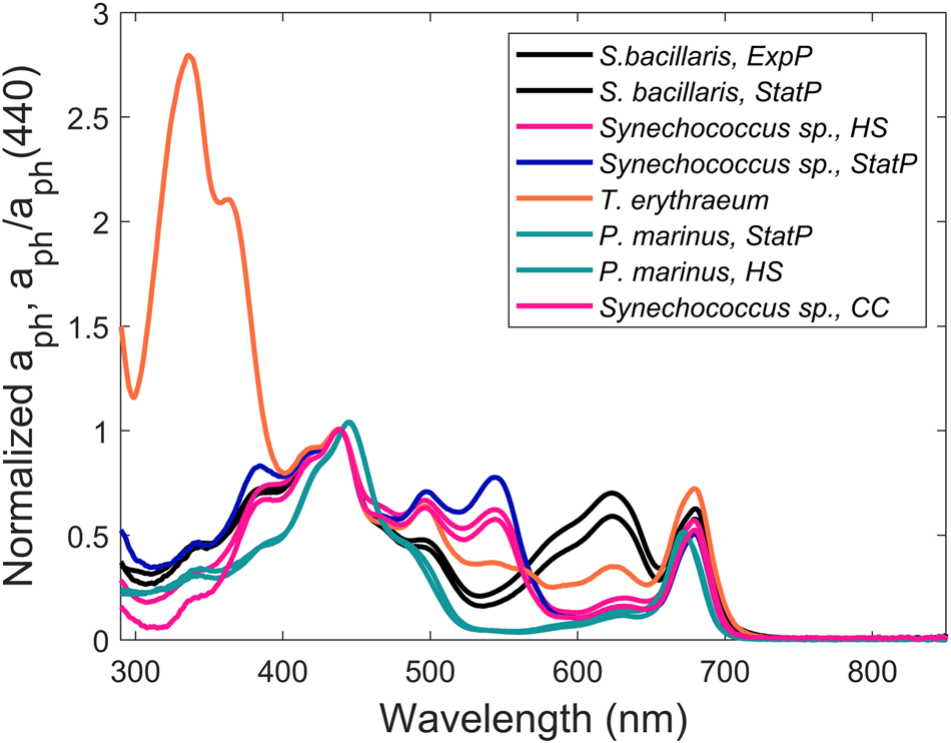
Phytoplankton absorption, a_ph_(λ) normalized to a_ph_(440), of cyanobacterial species measured in this study. ExpP = Exponential phase. StatP = Stationary phase. CC = Climate change. HS = HyperSAS.

The compiled dataset represents a wide diversity of strains, collected from around the world’s oceans, and highlights the challenges of describing an ‘average diatom or dinoflagellate or cyanobacteria’ (Fig. 5). Rather than focus on this challenge, our diverse dataset can allow users to ‘regionalize’ their analysis by choosing a subset of strains that may be more representative of the focal region, as it is becoming increasingly well recognized that ‘global parameter sets’ perform poorly when downscaled to specific regions (e.g., coastal zone vs. oceanic zone). As an example of the usage of this dataset, the two species of *Synechococcus* sp. (CCMP1334) and *Synechococcus bacillaris* (CCMP1333) can be distinguished by the differential expression of the pigments phycoerythrin (PE) and phycocyanin (PC). The differential expression of the phycobilins is determined by the quality of light field in the water column^58^. PE is commonly expressed by species found in clear waters from which *Synechococcus* sp. (CCMP1334) was isolated and where blue wavelengths of light are not strongly absorbed. *T. erythraeum* also expresses both PE and PC. A small PE peak can be observed in the absorption spectrum of CCMP1985, which was isolated from the North Atlantic^59,60^. In contrast, PC is expressed in species found in more turbid, coastal waters, from which *S. bacillaris* was isolated, where blue light is more strongly absorbed. *Microcystis aeruginosa* (CCMP3462) also shows a PC absorption peak and was isolated in the turbid waters of Lake Erie. Furthermore, the wide diversity of parameters measured for each algal strain expands its utility when attempting to validate remotely sensed PCC models against direct field measurements that are not always uniformly measured.

## Supplementary information

Supplemental Table 1

## Data Availability

No custom code was generated as part of this publication or required for use of the data.
