# Supplementary material for: Phytoplankton optical fingerprint libraries for development of phytoplankton ocean color satellite products
Source: Sci Data. 2024 Feb 3;11:168. doi: 10.1038/s41597-024-03001-z (PMC11422499; doi:10.1038/s41597-024-03001-z)
Supplement: Supplementary file 1 — Supplemental Table 1 [file 41597_2024_3001_MOESM1_ESM.docx]

**Supplemental Table 1**. Complete list of phytoplankton strains studied and experiments for which data was collected. Additional details on each strain can be found in Neeley *et al.* (2022). Gray boxes denote the growth phase (Exponential=Exp, Stationary=Stat), mixed culture (mc), climate change (CC) and evolution (Evo) experiments where a specific strain was examined. For mixed culture experiments, the species mixed are determined by reading down each experimental column, e.g., in mc1, *Thalassiosira oceanica, Prochlorococcus marinus, Tetraselimis sp.* and *Emiliania huxleyi* were mixed together. The number in a gray box denotes the sampling event in which a measurement was made. Sampling event numbers are as follows: 1 – October 11-20, 2017; 2 – December 6-15, 2017; 3 – February 26 – March 10, 2018; 4 – April 29 – May 10, 2018; 5 – February 12-14, 2019.

| **Strain Name** | **Exp** | **Stat** | **mc1** | **mc2** | **mc3** | **mc4** | **mc5** | **mc6** | **mc7** | **mc8** | **CC** | **Evo** |
| --- | --- | --- | --- | --- | --- | --- | --- | --- | --- | --- | --- | --- |
| **Diatoms** |  |  |  |  |  |  |  |  |  |  |  |  |
| *Amphora coffeaeformis* (CCMP127) | 3 | 3 |  |  |  |  |  |  |  |  |  |  |
| *Chaetoceros diadema* (CCMP3261) | 4 |  |  |  |  |  |  |  |  |  |  |  |
| *Chaetoceros muelleri* (CCMP1316) | 2 | 2 |  |  |  |  |  | 2 |  | 2 |  |  |
| *Chaetoceros socialis* (CCMP3263) | 4 | 4 |  |  |  |  |  |  |  |  |  |  |
| *Corethron hystrix* (CCMP308) | 4 |  |  |  |  |  |  |  |  |  |  |  |
| *Ditylum brightwellii* (CCMP359) |  |  |  |  |  |  |  |  |  |  |  | 5 |
| *Ditylum brightwellii* (CCMP2227) |  |  |  |  |  |  |  |  |  |  |  | 5 |
| *Ditylum brightwellii* (CCMP3369) |  |  |  |  |  |  |  |  |  |  |  | 5 |
| *Fragilariopsis cylindrus* (CCMP3323) | 3 |  |  |  |  |  |  |  |  |  |  |  |
| *Grammonema striatula* (CCMP1094) | 3 | 3 |  |  |  |  |  |  |  |  |  |  |
| *Nitzschia sp.* (CCMP580) | 3 |  |  |  |  |  |  |  |  |  |  |  |
| *Phaeodactylum tricornutum* (CCMP632) | 3 |  |  |  |  |  |  |  |  |  |  |  |
| *Rhizoselenia setigera* (CCMP1330) | 4 |  |  |  |  |  |  |  |  |  |  |  |
| *Thalassiosira guillardii* (CCMP988) | 2 | 2 |  |  |  |  |  | 2 | 2 |  |  |  |
| *Thalassiosira nordenskioeldii* (CCMP1017) | 2 |  |  |  |  |  |  |  |  |  |  |  |
| *Thalassiosira oceanica* (CCMP1005) | 1 | 1 | 1 |  | 1 | 1 | 1 |  |  |  | 4 |  |
| **Chlorarachniophyte** |  |  |  |  |  |  |  |  |  |  |  |  |
| *Bigelowiella natans* (CCMP2755) | 2 | 2 |  |  |  |  |  |  |  |  |  |  |
| **Cyanobacteria** |  |  |  |  |  |  |  |  |  |  |  |  |
| *Microcystis aeruginosa* (CCMP3462) | 4 |  |  |  |  |  |  |  |  |  |  |  |
| *Prochlorococcus marinus* (CCMP2389) | 1 | 1 | 1 | 1 |  |  | 1 |  |  |  |  |  |
| *Synechococcus bacillaris* (CCMP1333) | 3 | 3 |  |  |  |  |  |  |  |  |  |  |
| *Synechoccoccus* sp. (CCMP1334) | 1 | 1 |  | 1 | 1 |  | 1 |  |  |  | 4 |  |
| *Trichodesmium erythraeum* (CCMP1985) | 4 |  |  |  |  |  |  |  |  |  |  |  |
| **Cryptophyte** |  |  |  |  |  |  |  |  |  |  |  |  |
| *Rhodomonas salina* (CCMP1319) | 4 | 4 |  |  |  |  |  |  |  |  |  |  |
| **Dinoflagellates** |  |  |  |  |  |  |  |  |  |  |  |  |
| *Amphidinium cartarae* (CCMP119) |  |  |  |  |  |  |  |  |  |  |  | 5 |
| *Amphidinium cartarae* (CCMP2400) |  |  |  |  |  |  |  |  |  |  |  | 5 |
| *Amphidinium gibbosum* (CCMP120) |  | 3 |  |  |  |  |  |  |  |  |  |  |
| *Alexandrium tamarense* (CCMP1771) |  | 3 |  |  |  |  |  |  |  |  |  |  |
| *Heterocapsa arctica* (CCMP445) | 3 |  |  |  |  |  |  |  |  |  |  |  |
| *Karenia brevis* (CCMP2281) | 3 | 3 |  |  |  |  |  |  |  |  |  |  |
| *Prorocentrum lima* (CCMP3310) |  | 3 |  |  |  |  |  |  |  |  |  |  |
| *Prorocentrum minimum* (CCMP1329) | 3 | 3 |  |  |  |  |  |  |  |  |  |  |
| **Pelagophyte** |  |  |  |  |  |  |  |  |  |  |  |  |
| *Pelagomonas calceolate* (CCMP1756) | 1 | 1 |  | 1 |  |  | 1 |  |  |  |  |  |
| **Prasinophytes** |  |  |  |  |  |  |  |  |  |  |  |  |
| *Ostreococcus lucimarinus* (CCMP3430) | 3 | 3 |  |  |  |  |  |  |  |  | 4 |  |
| *Prasinococcus capsulatus* (CCMP1194) | 2 | 2 |  |  |  |  |  |  |  | 2 |  |  |
| *Pyramimonas parkae* (CCMP725) | 4 | 4 |  |  |  |  |  |  |  |  |  |  |
| *Tetraselmis sp.* (CCMP908) | 1 | 1 | 1 |  |  |  | 1 |  |  |  |  |  |
| **Haptophytes** |  |  |  |  |  |  |  |  |  |  |  |  |
| *Chrysotila stipitate* (CCMP3382) | 3 |  |  |  |  |  |  |  |  |  |  |  |
| *Cruciplacolithus neohelis* (CCMP298) |  | 3 |  |  |  |  |  |  |  |  |  |  |
| *Emiliania huxleyi* (CCMP371) | 3 | 3 |  |  |  |  |  |  |  |  |  |  |
| *Emiliania huxleyi* (CCMP373) | 1 | 1 | 1 |  |  | 1 | 1 |  |  |  |  |  |
| *Emiliania huxleyi* (CCMP1949) | 2 | 2 |  |  |  |  |  |  | 2 |  |  |  |
| *Isochrysis galbana* (CCMP1323) |  | 3 |  |  |  |  |  |  |  |  |  |  |
| *Phaeocystis Antarctica* (CCMP3314) | 2 | 2 |  |  |  |  |  |  |  |  |  |  |
| *Phaeocystis globose* (CCMP1805) | 3 | 3 |  |  |  |  |  |  |  |  |  |  |
| *Pleurochrysis carterae* (CCMP647) | 2 | 2 |  |  |  |  |  | 2 |  |  |  |  |
| *Prymnesium parvum* (CCMP708) |  | 3 |  |  |  |  |  |  |  |  |  |  |
| *Prymnesium polylepis* (CCMP1757) | 4 | 4 |  |  |  |  |  |  |  |  |  |  |
| **Raphidophytes** |  |  |  |  |  |  |  |  |  |  |  |  |
| *Heterosigma akashiwo* (CCMP452) |  |  |  |  |  |  |  |  |  |  |  | 5 |
| *Heterosigma akashiwo* (CCMP1680) |  |  |  |  |  |  |  |  |  |  |  | 5 |
| *Heterosigma akashiwo* (CCMP2393) |  |  |  |  |  |  |  |  |  |  |  | 5 |
| *Heterosigma akashiwo* (CCMP3374) |  |  |  |  |  |  |  |  |  |  |  | 5 |
